# Supplementary material for: Qualitative Analysis of Multiple Phytochemical Compounds in Tojapride Based on UHPLC Q-Exactive Orbitrap Mass Spectrometry
Source: Molecules. 2022 Oct 6;27(19):6639. doi: 10.3390/molecules27196639 (PMC9571116; doi:10.3390/molecules27196639)
Supplement: Supplementary file 1 [file molecules-27-06639-s001.zip › molecules-1944347-supplementary.pdf]

Supplementary Table S1: Retention times and mass spectral data of Tojapride.

Peak	t_R	Theoretical Mass *m/z*	Experimental Mass *m/z*	Error (ppm)	Formula	MS/MS fragment (-)	MS/MS fragment (+)	Identification
1	0.88*	191.0561	191.0553	-4.25	$C_7H_{12}O_6$	MS^2[191]: 111.0075(100), 87.0074(52), 85.0282(46)		Quinic acid
2	0.94*	133.0142	133.0132	-7.87	$C_4H_6O_5$	MS^2[133]: 115.9657(100), 71.0125(59), 133.0132(31)		Malic Acid
3	1.29	331.0671	331.0674	1.03	$C_{13}H_{16}O_{10}$	MS^2[331]: 169.0134(100), 125.0233(83)		1-Galloyl-β-glucose isomer
4	1.46*	129.0193	129.0183	-8.00	$C_5H_6O_4$	MS^2[129]: 85.0282(100)		Citraconic acid
5	1.46*	169.0142	169.0135	-4.65	$C_7H_6O_5$	MS^2[169]: 125.0232(100)		Gallic acid
6	1.96	197.0455	197.0450	-2.77	$C_9H_{10}O_5$	MS^2[197]: 179.03429(100), 152.8941(4)		alpha-(3,4-dihydroxyphenyl) lactic acid
7	2.05	331.0671	331.0675	1.42	$C_{13}H_{16}O_{10}$	MS^2[331]: 169.0134(100), 125.0233(80)		1-Galloyl-β-glucose isomer
8	2.42*	153.0193	153.0184	-6.09	$C_7H_6O_4$	MS^2[153]: 109.0283(100)		Protocatechuic acid
9	3.08*	353.0878	353.0881	0.84	$C_{16}H_{18}O_9$	MS^2[353]: 191.0554(100), 135.0441(73), 179.0342(52)		Chlorogenic acid
10	4.03	153.0193	153.0184	-5.96	$C_7H_6O_4$	MS^2[153]: 109.0283(100)		Protocatechuic acid isomer
11	4.24*	183.0300	183.029	-3.53	$C_8H_8O_5$	MS^2[183]: 140.0105(100),		Methyl gallate

						124.0154(33), 168.0054(10), 111.0074(5)		
12	5.57	153.0193	153.0184	-5.89	$C_7H_6O_4$	MS^2[153]: 109.0283(100)		Protocatechuic acid isomer
13	6.11	193.0506	193.0499	-3.74	$C_{10}H_{10}O_4$	MS^2[193]: 134.0362(100), 178.0265(29), 149.0597(18)		Isoferulic acid isomer
14	6.29*	353.0878	353.0881	0.84	$C_{16}H_{18}O_9$	MS^2[353]:191.0554(100), 135.0441(13), 179.0342(8)		Cryptochlorogenic acid
15	6.37*	179.0350	179.0343	-3.98	$C_9H_8O_4$	MS^2[179]: 135.0441(100)		Caffeic acid
16	7.53	193.0506	193.0500	-3.17	$C_{10}H_{10}O_4$	MS^2[193]: 135.0441(100), 134.0362(17), 178.0267(9)		Isoferulic acid isomer
17	8.34	771.1989	771.2001	1.55	$C_{33}H_{40}O_{21}$	MS^2[771]: 301.0359(100), 300.0273(27),463.0892(10), 609.1465(6)		Quercetin-O-glucoside-rutinoside
18	8.80*	173.0819	173.0812	-4.52	$C_8H_{14}O_4$	MS^2[173]: 93.033(100), 111.0803(41), 83.0489(8)		Octanedioic acid
19	9.03	193.0506	193.0501	-2.86	$C_{10}H_{10}O_4$	MS^2[193]:149.0599(100), 134.0363(10), 178.0265(2)		Isoferulic acid isomer
20	9.24	593.1512	593.1519	1.23	$C_{27}H_{30}O_{15}$	MS^2[593]: 353.0676(100), 297.0769(59), 383.0773(52), 473.1092(21)		Vicenin 2 isomer
21	9.54*	342.1705	342.1697	-0.98	$C_{20}H_{24}NO_4^+$		MS^2[342]: 265.0855(100), 297.1118(95),	Magnoflorine

							237.0907(40), 282.0883(36)	
22	9.87	741.2248	741.2255	1.04	$C_{33}H_{42}O_{19}$	MS^2[741]: 271.0613(100), 151.0027(64), 119.0491(32)		Naringenin 4`-O-glucoside 7-O-rutinoside isomer
23	10.37*	137.0244	137.0235	-6.62	$C_7H_6O_3$	MS^2[137]: 93.0333(100)		Salicylic acid
24	10.48	593.1512	593.1521	1.53	$C_{27}H_{30}O_{15}$	MS^2[593]: 473.1092(100), 353.0672(64), 383.0777(36)		Vicenin 2 isomer
25	10.48	609.1461	609.1471	1.61	$C_{27}H_{30}O_{16}$	MS^2[609]: 285.0406(100), 447.0940(85)		Luteolin-O-Lactoside
26	10.51	741.2248	741.2259	1.54	$C_{33}H_{42}O_{19}$	MS^2[741]: 151.0027(100), 119.0490(34), 271.0613(52)		Naringenin 4`-O-glucoside 7-O-rutinoside isomer
27	10.60	771.1989	771.2003	1.80	$C_{33}H_{40}O_{21}$	MS^2[771]: 463.0854(100), 609.1464(54), 301.0355(20)		Quercetin-O-glucoside-rutinoside
28	10.83*	447.0933	447.0937	0.84	$C_{21}H_{20}O_{11}$	MS^2[447]: 327.0514(100), 357.0619(23), 297.0406(13)		Orientin
29	10.89	609.1461	609.1471	1.61	$C_{27}H_{30}O_{16}$	MS^2[609]: 447.0937(100), 285.0406(22)		Luteolin-O-Lactoside
30	10.92	417.1191	417.1197	1.40	$C_{21}H_{22}O_9$	MS^2[417]: 119.0491(100), 255.0662(70), 135.0077(28), 153.0183(23)		Isoliquiritin isomer
31	11.08	505.2306	505.2228	0.06	$C_{26}H_{34}NO_9^+$		MS^2[505]: 342.1698(100)	Magnoflorine-O-glucoside

32	11.18	447.0933	447.0938	1.18	$C_{21}H_{20}O_{11}$	MS^2[447]: 327.0514(100), 357.0620(62), 297.0410(19)		Orientin isomer
33	11.27	593.1512	593.1519	1.23	$C_{27}H_{30}O_{15}$	MS^2[593]: 473.1094(100), 353.0672(80), 383.0777(75)		Vicenin 2 isomer
34	11.40	471.2013	471.2012	-0.31	$C_{26}H_{30}O_{8}$		MS^2[471]: 161.0596(100), 425.1975(36), 367.1907(11)	Limonin isomer
35	11.50	549.1614	549.1620	1.14	$C_{26}H_{30}O_{13}$	MS^2[549]: 255.0662(100), 119.0491(88), 135.0078(51), 153.0184(33)		Liquiritin apioside isomer
36	11.56*	417.1191	417.1195	0.83	$C_{21}H_{22}O_{9}$	MS^2[417]: 255.0662(100), 135.0077(53), 119.0491(42), 153.0184(25)		Liquiritin
37	11.70	389.1242	389.1245	0.74	$C_{20}H_{22}O_{8}$	MS^2[389]: 227.0711(100)		Polydatin
38	11.76	771.2353	771.2364	1.39	$C_{34}H_{44}O_{20}$	MS^2[771]: 301.0720(100)		Hesperidin 7-O-glucoside isomer
39	11.77	342.1705	342.1700	-0.10	$C_{20}H_{24}NO_4^+$		MS^2[342]: 265.0850(100), 297.1124(86), 282.0883(32), 237.0908(29)	Magnoflorine isomer
40	11.79	741.2248	741.2258	1.45	$C_{33}H_{42}O_{19}$	MS^2[741]: 151.0028(100), 271.0614(41), 119.0491(37)		Naringenin 4`-O-glucoside 7-O-rutinoside isomer

41	11.93	593.1512	593.1519	1.12	$C_{27}H_{30}O_{15}$	MS^2[593]: 473.1091(100), 353.0669(68), 383.0772(66)		Vicenin 2 isomer
42	11.96*	431.0984	431.0988	1.04	$C_{21}H_{20}O_{10}$	MS^2[431]: 311.0564(100), 283.0612(30), 341.0665(7)		Vitexin
43	12.11	609.1461	609.1458	-0.59	$C_{27}H_{30}O_{16}$	MS^2[609]: 447.0934(100), 285.0406(40)		Luteolin-O-Lactoside
44	12.16	549.1614	549.1622	1.47	$C_{26}H_{30}O_{13}$	MS^2[549]: 119.0491(100), 255.0663(93), 135.0078(74), 153.0185(46)		Liquiritin apioside isomer
45	12.31	263.1288	263.1292	1.02	$C_{15}H_{20}O_4$	MS^2[263]: 219.1388(100), 201.1280(14), 204.1151(9)		Abscisic acid isomer
46	12.42	255.0663	255.0657	-2.32	$C_{15}H_{12}O_4$	MS^2[255]: 119.0488(100), 135.0074(28), 153.0181(13)		Liquiritigenin isomer
47	12.48	741.2248	741.2263	2.03	$C_{33}H_{42}O_{19}$	MS^2[741]: 151.0028(100), 119.0491(36), 271.0615(46)		Naringenin 4`-O-glucoside 7-O-rutinoside isomer
48	12.50	342.1705	342.1700	-0.10	$C_{20}H_{24}NO_4^+$		MS^2[342]: 265.0864(100), 297.1119(54), 237.0903(23)	Magnoflorine isomer
49	12.66	463.0882	463.0892	2.23	$C_{21}H_{20}O_{12}$	MS^2[463]: 301.0355(100), 300.0275(15), 151.0027(14)		Hyperoside isomer

50	12.72	609.1461	609.1473	1.92	$C_{27}H_{30}O_{16}$	MS^2[609]: 301.0355(100), 151.0026(17), 300.0272(5)		Rutin isomer
51	12.75	447.0933	447.0938	1.04	$C_{21}H_{20}O_{11}$	MS^2[447]: 285.0406(100)		Luteolin 7-O-β-D-glucoside isomer
52	12.80	593.1512	593.1526	2.36	$C_{27}H_{30}O_{15}$	MS^2[593]: 383.0772(100), 353.0669(68), 473.1091(65)		Vicenin 2 isomer
53	12.95*	463.0882	463.0890	1.64	$C_{21}H_{20}O_{12}$	MS^2[463]: 300.0277(100), 301.0353(81), 151.0027(10)		Hyperoside
54	12.95*	193.0506	193.0502	-2.29	$C_{10}H_{10}O_4$	MS^2[193]: 161.0236(100), 134.0362(63), 178.0268(18), 149.0597(10)		Isoferulic acid
55	13.07*	609.1461	609.1475	2.22	$C_{27}H_{30}O_{16}$	MS^2[609]: 300.0216(100), 271.0249(65), 301.0361(39), 151.0031(17)		Rutin
56	13.07	431.0984	431.0990	1.53	$C_{21}H_{20}O_{10}$	MS^2[431]: 311.0564(100), 341.0669(35), 283.0613(25)		Vitexin isomer
57	13.08	342.1705	324.1230	-0.05	$C_{20}H_{24}NO_4^+$		MS^2[342]: 265.0860(100), 237.0909(10)	Magnoflorine isomer
58	13.18	771.2353	771.2368	1.95	$C_{34}H_{44}O_{20}$	MS^2[771]: 301.0720(100), 151.0027(37), 286.0476(17), 242.0591(14)		Hesperidin 7-O-glucoside isomer

59	13.18*	187.0976	187.0971	-2.79	$C_9H_{16}O_4$	MS^2[187]:125.0161(100), 187.0970(50), 97.0647(11)	Azelaic acid
60	13.30*	463.0882	463.0892	2.16	$C_{21}H_{20}O_{12}$	MS^2[463]: 300.0277(100), 301.0352(58)	Isoquercitrin
61	13.42	609.1461	609.1475	2.22	$C_{27}H_{30}O_{16}$	MS^2[609]: 301.0356(100), 300.0279(41),151.0027(10)	Rutin isomer
62	13.42	771.2353	771.2369	2.03	$C_{34}H_{44}O_{20}$	MS^2[771]: 301.0720(100), 151.0027(32), 286.0482(18), 242.0578(17)	Hesperidin 7-O-glucoside isomer
63	13.56*	447.0933	447.0934	0.21	$C_{21}H_{20}O_{11}$	MS^2[447]: 285.0406(100), 151.0026(6), 133.0283(6)	Luteolin 7-O-β-D-glucoside
64	13.56*	593.1512	579.1726	1.18	$C_{27}H_{30}O_{15}$	MS^2[593]: 284.0332(100), 151.0030(4), 107.0126(2)	Kaempferol 3-O-neohesperidoside
65	13.62	579.1719	579.1724	0.75	$C_{27}H_{32}O_{14}$	MS^2[579]: 271.0615(100), 151.0028(67), 119.0491(43)	Narirutin isomer
66	13.68	461.0725	461.0731	1.11	$C_{21}H_{18}O_{12}$	MS^2[461]: 285.0406(100), 133.0284(8), 151.0030(3), 199.0397(2)	Luteolin 7-O-glucuronide
67	13.73	271.0612	271.0614	0.86	$C_{15}H_{12}O_5$	MS^2[271]: 119.0491(100), 151.0028(78), 177.0186(8)	Naringenin isomer

68	13.76	433.1140	433.1144	0.97	$C_{21}H_{22}O_{10}$	MS^2[433]: 271.0616(100), 151.0028(53), 119.0492(52)		Naringenin 7-O-glucoside isomer
69	13.88	771.2353	771.2371	2.26	$C_{34}H_{44}O_{20}$	MS^2[771]: 301.0721(100), 151.0028(58(58),242.0585(16), 286.0495(15)		Hesperidin 7-O-glucoside isomer
70	14.08	263.1288	263.1293	1.47	$C_{15}H_{20}O_4$	MS^2[263]: 219.1387(100), 201.1278(6), 204.1148(4)		Abscisic acid isomer
71	14.17	593.1512	579.1729	1.59	$C_{27}H_{30}O_{15}$	MS^2[593]: 284.0308(100), 151.0029(6),107.0128(4)		Kaempferol 3-O-neohesperidoside isomer
72	14.17	741.2248	741.2263	2.03	$C_{33}H_{42}O_{19}$	MS^2[741]: 151.0028(100), 271.0617(75), 119.0491(44)		Naringenin 4`-O-glucoside 7-O-rutinoside isomer
73	14.23	433.1140	433.1144	0.97	$C_{21}H_{22}O_{10}$	MS^2[433]: 271.0614(100), 151.0028(57), 119.0491(41)		Naringenin 7-O-glucoside isomer
74	14.30	324.1236	324.1226	-1.28	$C_{19}H_{18}NO_4^+$		MS^2[324]: 309.0992(100), 280.0966(92), 266.0809(29), 294.0757(26)	Demethyleneberberine
75	14.37	271.0612	271.0614	0.86	$C_{15}H_{12}O_5$	MS^2[271]: 119.0491(100), 151.0027(35), 177.0186(5)		Naringenin isomer
76	14.40	579.1719	579.1724	0.75	$C_{27}H_{32}O_{14}$	MS^2[579]: 151.0028(100),		Narirutin isomer

						271.0614(51), 119.0491(41)		
77	14.55	322.1079	322.1072	-0.64	$C_{19}H_{16}NO_4^+$		MS^2[322]: 307.0837(100), 279.0887(37)	Groenlandicine
78	14.71	741.2248	741.22632	2.12	$C_{33}H_{42}O_{19}$	MS^2[741]: 151.0028(100), 271.0616(58), 119.0492(43)		Naringenin 4`-O-glucoside 7-O-rutinoside isomer
79	14.97*	593.1512	593.1524	2.03	$C_{27}H_{30}O_{15}$	MS^2[593]: 285.0406(100)		Nicotiflorin
80	15.09	447.0933	447.0941	1.73	$C_{21}H_{20}O_{11}$	MS^2[447]: 285.0408(100)		Luteolin 7-O-β-D-glucoside isomer
81	15.23*	609.1825	609.1834	1.41	$C_{28}H_{34}O_{15}$	MS^2[609]:301.0720(100), 151.0027(16), 286.0488(10)		Hesperidin
82	15.27	324.1236	324.1228	-0.63	$C_{19}H_{18}NO_4^+$		MS^2[324]: 309.0993(100), 294.0759(73), 266.0809(22), 280.0967(6)	Demethyleneberberine
83	15.27	338.1392	338.1384	-0.75	$C_{20}H_{20}NO_4^+$		MS^2[338]: 322.1071(100), 308.0916(10), 294.1120(4)	Columbamine
84	15.37	301.0354	301.0360	2.11	$C_{15}H_{10}O_7$	MS^2[301]: 151.0027(100), 107.0126(40)		Quercetin isomer
85	15.52	771.2353	771.2367	1.78	$C_{34}H_{44}O_{20}$	MS^2[771]: 301.0719(100)		Hesperidin 7-O-glucoside isomer
86	15.55	593.1512	593.1522	1.73	$C_{27}H_{30}O_{15}$	MS^2[593]: 285.04065(100)		Nicotiflorin isomer
87	15.63	301.0718	301.0721	0.96	$C_{16}H_{14}O_6$	MS^2[301]: 151.0027(100), 286.0484(33)		Hesperetin isomer

88	15.63	463.1246	463.1248	2.74	$C_{22}H_{24}O_{11}$	MS^2[463]: 301.0720(100), 151.0028(13), 286.0483(9), 242.0584(7)		Hesperetin 7-O-β-D-glucuronide
89	15.63	431.0984	431.0990	1.39	$C_{21}H_{20}O_{10}$	MS^2[431]: 269.0456(100)		Aloe Emodin 8-glucoside isomer
90	15.85	324.1236	324.1228	-0.63	$C_{19}H_{18}NO_4^+$		MS^2[324]: 309.0994(100), 280.0967(20), 294.0759(9), 266.0814(4)	Demethyleneberberine
91	15.97	609.1825	609.1830	0.80	$C_{28}H_{34}O_{15}$	MS^2[609]: 301.0721(100), 151.0028(30), 286.0484(21)		Neohesperidin
92	16.20	445.1140	445.1145	1.08	$C_{22}H_{22}O_{10}$	MS^2[445]: 283.0613(100), 268.0378(10), 269.0421(1)		Oroxylin A-O-glucoside
93	16.20	771.2353	771.2366	1.70	$C_{34}H_{44}O_{20}$	MS^2[771]: 301.0719(100), 151.0026(28), 286.0469(21), 242.0580(12)		Hesperidin 7-O-glucoside isomer
94	16.32	623.1618	623.1627	1.54	$C_{28}H_{32}O_{16}$	MS^2[623]: 315.0514(100), 300.0276(72), 151.0027(12), 271.0252(5)		Methyl-quercetin-O-rutinoside
95	16.57	445.1140	445.1146	1.35	$C_{22}H_{22}O_{10}$	MS^2[445]: 283.0615(100), 269.0451(17), 268.0383(16)		Oroxylin A-O-glucoside
96	16.60*	263.1288	263.1293	1.47	$C_{15}H_{20}O_4$	MS^2[263]: 219.1386(100),		Abscisic acid

						204.1150(82), 201.1279(29)		
97	16.69	621.1825	621.1835	1.70	$C_{29}H_{34}O_{15}$	MS^2[621]: 151.0027(100), 119.0491(64), 271.0612(64(64)		Naringenin derivative
98	16.73	322.1079	322.1072	-0.73	$C_{19}H_{16}NO_4^+$		MS^2[322]: 307.0837(100), 279.0888(18)	Groenlandicine
99	16.89	623.1618	623.1628	1.74	$C_{28}H_{32}O_{16}$	MS^2[623]: 300.0276(100), 315.0512(85), 151.0028(19), 271.0253(6)		Methyl-quercetin-O-rutinoside
100	16.90	320.0923	320.0916	-0.29	$C_{19}H_{14}NO_4^+$		MS^2[320]:292.0966(100), 277.0732(46), 262.0859(43), 234.0912(22)	Coptisine
101	16.92*	287.0561	287.0565	1.42	$C_{15}H_{12}O_6$	MS^2[287]: 135.0441(100), 107.0126(23), 151.0027(21)		Eriodictyol
102	16.93	338.1392	338.1384	-0.84	$C_{20}H_{20}NO_4^+$		MS^2[338]: 322.1072(100), 294.1122(73), 308.0915(45)	Columbamine
103	17.00	621.1825	621.1834	1.48	$C_{29}H_{34}O_{15}$	MS^2[621]: 151.0027(100), 271.0614(59), 119.0491(57)		Naringenin derivative
104	17.16	336.1240	336.1230	-0.13	$C_{20}H_{18}NO_4^+$		MS^2[336]: 320.0916(100), 292.0965(37), 321.0995(13), 278.1816(1)	Berberine isomer

105	17.20	459.1297	459.13034	1.46	$C_{23}H_{24}O_{10}$	MS^2[459]: 255.0663(100), 135.0077(35), 153.0184(26), 119.0490(20)		6'-Acetylliquiritin isomer
106	17.22	338.1392	338.1384	-0.75	$C_{20}H_{20}NO_4^+$		MS^2[338]: 322.1071(100), 294.1122(70), 308.0913(43)	Columbamine
107	17.46	431.0984	431.0989	1.32	$C_{21}H_{20}O_{10}$	MS^2[431]: 269.0459(100)		Aloe Emodin 8-glucoside isomer
108	17.53	320.0923	320.0918	0.21	$C_{19}H_{14}NO_4^+$		MS^2[320]: 292.0966(100), 277.0732(41), 262.0860(34), 234.0915(18)	Coptisine
109	17.55	621.1825	621.1837	1.86	$C_{29}H_{34}O_{15}$	MS^2[621]: 151.0027(100), 271.0613(64), 119.0490(56)		Naringenin derivative
110	17.98	549.1614	549.1621	1.36	$C_{26}H_{30}O_{13}$	MS^2[549]: 119.0491(100), 255.0663(88), 135.0078(65), 153.0184(49)		Liquiritin apioside isomer
111	18.01*	445.0776	445.07809	1.02	$C_{21}H_{18}O_{11}$	MS^2[445]:269.0457(100), 161.0447(4)		Baicalin
112	18.12*	417.1191	417.1195	0.97	$C_{21}H_{22}O_9$	MS^2[417]: 255.0662(100), 135.0077(53), 119.0491(42), 153.0184(25)		Isoliquiritin
113	18.18	579.1719	579.1726	1.18	$C_{27}H_{32}O_{14}$	MS^2[579]: 151.0027(100), 271.0613(52), 119.0491(46)		Narirutin isomer

114	18.40	338.1392	338.1385	-0.58	$C_{20}H_{20}NO_4^+$		MS^2[338]: 322.1071(100), 294.1118(26), 308.1177(5)	Columbamine
115	18.50	549.1614	549.1620	1.14	$C_{26}H_{30}O_{13}$	MS^2[549]: 255.0662(100), 119.0491(84), 135.0077(53), 153.0185(34)		Liquiritin apioside isomer
116	18.54	322.1079	322.1072	-0.73	$C_{19}H_{16}NO_4^+$		MS^2[322]: 307.0836(100), 279.0889(19)	Groenlandicine
117	18.66	352.1550	352.1541	-0.55	$C_{21}H_{22}NO_4^+$		MS^2[352]: 336.1226(100), 308.1277(66), 294.1122(35), 322.1069(30)	Palmatine
118	18.73	417.1191	417.1195	0.97	$C_{21}H_{22}O_9$	MS^2[417]: 119.0491(100), 255.0662(77), 135.0078(30), 153.0184(25)		Isoliquiritin isomer
119	19..81	285.0768	285.0766	-0.87	$C_{16}H_{14}O_5$	MS^2[285]: 164.0106(100), 151.0028(60)		Sakuranetin isomer
120	19.17	255.0663	255.0657	-2.32	$C_{15}H_{12}O_4$	MS^2[255]: 119.0488(100), 135.0074(28), 153.0181(13)		Liquiritigenin isomer
121	19.47	336.1240	336.1225	0.98	$C_{20}H_{18}NO_4^+$		MS^2[336]: 320.0914(100), 292.0964(91), 321.0994(51), 278.0810(35)	Berberine isomer
122	19.49*	283.0612	283.0613	0.51	$C_{16}H_{12}O_5$	MS^2[283]: 268.0379(100),		Calycosin

						211.0395(20), 239.0354(10)		
123	19.54*	301.0354	301.0355	0.48	$C_{15}H_{10}O_7$	MS^2[301]: 151.0028(100), 107.0127(56)		Quercetin
124	19.69	609.1825	609.1832	1.21	$C_{28}H_{34}O_{15}$	MS^2[609]: 301.0721(100), 151.0028(32), 286.0485(19), 242.0582(15)		Hesperidin isomer
125	19.78	593.1876	593.1877	0.27	$C_{28}H_{34}O_{14}$	MS^2[593]: 285.0769(100), 164.0107(17), 151.00279(11)		Poncirin isomer
126	19.97	352.1550	352.1540	-0.98	$C_{21}H_{22}NO_4^+$		MS^2[352]: 336.1227(100), 308.1277(64), 294.1123(38), 322.1071(31)	Palmatine
127	20.10*	285.0405	285.0407	0.98	$C_{15}H_{10}O_6$	MS^2[285]: 133.0186(100), 151.0028(30), 175.0392(18), 199.0394(11)		Luteolin
128	20.21	621.1825	621.1832	1.19	$C_{29}H_{34}O_{15}$	MS^2[621]: 151.0027(100), 271.0614(55), 119.0491(45)		Naringenin derivative
129	20.37	336.1240	336.1229	-0.52	$C_{20}H_{18}NO_4^+$		MS^2[336]: 320.0915(100), 292.0965(89), 321.0994(55), 278.0810(32)	Berberine isomer
130	20.53	593.1876	593.1877	0.27	$C_{28}H_{34}O_{14}$	MS^2[593]: 285.0769(100),		Poncirin isomer

						151.0028(20), 164.0106(16)	
131	20.56*	271.0612	271.0613	0.42	$C_{15}H_{12}O_5$	MS^2[271]: 119.0491(100), 151.0027(76), 177.0186((10), 227.0710(1)	Naringenin
132	21.02	621.1825	621.1832	1.09	$C_{29}H_{34}O_{15}$	MS^2[621]: 151.0027(100), 119.0491(49), 271.0614(38)	Naringenin derivative
133	21.08	285.0405	285.0406	0.45	$C_{15}H_{10}O_6$	MS^2[285]: 133.0284(100), 199.0394(30), 151.0027(11), 175.0392(6)	Luteolin isomer
134	21.11	431.0984	431.0985	0.33	$C_{21}H_{20}O_{10}$	MS^2[431]: 269.0458(100), 225.0555(18), 241.0458(4)	Aloe Emodin 8-glucoside isomer
135	21.83	853.3863	853.3886	2.59	$C_{42}H_{62}O_{18}$	MS^2[853]: 351.0570(100), 193.0347(20), 113.0229(14)	22-Hydroxyl-licorice saponin G2 isomer
136	21.86	263.1288	263.1292	1.36	$C_{15}H_{20}O_4$	MS^2[263]: 219.1388(100), 204.1152(15), 201.1280(6)	Abscisic acid isomer
137	22.32	301.0718	301.0720	0.66	$C_{16}H_{14}O_6$	MS^2[301]:151.0027(100), 286.0484(27)	Hesperetin isomer
138	22.49	853.3863	853.3872	0.95	$C_{42}H_{62}O_{18}$	MS^2[853]: 351.0577(100), 193.0348(13)	22-Hydroxyl-licorice saponin G2 isomer
139	22.96	853.3863	853.3881	2.02	$C_{42}H_{62}O_{18}$	MS^2[853]: 351.0569(100), 113.0250(16)	22-Hydroxyl-licorice saponin G2 isomer

140	23.25*	269.0455	269.0459	1.20	$C_{15}H_{10}O_5$	MS^2[269]: 117.0334(100), 151.0031(40), 225.0551(25), 241.0498(9)		Aloe emodin
141	23.36	419.1337	419.1330	-1.64	$C_{21}H_{22}O_9$		MS^2[419]: 389.0863(100), 404.1090(12), 371.0739(8)	3-Hydroxy-5,6,7,8,3',4'-Hexamethoxyflavone isomer
142	23.60	853.3863	853.3880	1.95	$C_{42}H_{62}O_{18}$	MS^2[853]: 351.0569(100)		22-Hydroxyl-licorice saponin G2 isomer
143	23.69	445.1140	445.1144	0.88	$C_{22}H_{22}O_{10}$	MS^2[445]: 283.0614(100), 240.0427(10), 268.0394(5)		Oroxylin A-O-glucoside
144	23.75	433.1140	433.1144	0.76	$C_{21}H_{22}O_{10}$	MS^2[433]: 283.0613(100), 239.0711(20)		Oroxylin A derivative
145	23.87	459.1297	459.1303	1.33	$C_{23}H_{24}O_{10}$	MS^2[459]: 255.0662(100), 153.0184(35),135.0077(35(35), 119.0490(18)		6'-Acetylliquiritin isomer
146	24.04	431.0984	431.0985	0.39	$C_{21}H_{20}O_{10}$	MS^2[431]: 269.0457(100), 225.0554(11), 241.0458(5)		Aloe Emodin 8-glucoside isomer
147	24.16	837.3914	837.3926	1.45	$C_{42}H_{62}O_{17}$	MS^2[837]: 113.0232(100), 193.0347(28), 351.0573(14)		Licorice-saponin G2 isomer
148	24.27	445.1140	445.1144	0.81	$C_{22}H_{22}O_{10}$	MS^2[445]: 283.0614(100), 240.0426(67), 239.0714(6), 268.0397(3)		Oroxylin A-O-glucoside

149	24.42	853.3863	853.3876	1.51	$C_{42}H_{62}O_{18}$	MS^2[853]: 113.0232(100), 193.0350(23), 351.0569(23)		22-Hydroxyl-licorice saponin G2 isomer
150	24.46	389.1231	389.1227	-0.91	$C_{20}H_{20}O_8$		MS^2[389]: 359.0758(100), 169.0131(33), 197.0078(12), 113.0236(4)	3-Hydroxy-5,6,7,8,4'-Pentamethoxyflavanone isomer
151	24.65	823.4122	823.4139	2.16	$C_{42}H_{64}O_{16}$	MS^2[823]: 113.0232(100), 193.0352(36), 351.0580(22)		Licorice saponin J2 isomer
152	24.91	837.3914	837.3933	2.18	$C_{42}H_{62}O_{17}$	MS^2[837]: 113.0236(100), 193.0347(39)		Licorice-saponin G2 isomer
153	25.10	419.1337	419.1335	-0.43	$C_{21}H_{22}O_9$		MS^2[419]: 389.0863(100), 371.0762(13), 404.1089(8)	3-Hydroxy-5,6,7,8,3',4'-Hexamethoxyflavone isomer
154	25.35	853.3863	853.3877	1.60	$C_{42}H_{62}O_{18}$	MS^2[853]: 351.0580(100)		22-Hydroxyl-licorice saponin G2 isomer
155	25.48	359.1125	359.1124	-0.28	$C_{19}H_{18}O_7$		MS^2[359]: 329.0653(100), 183.0288(14), 211.0236(12), 286.0466(11)	5-Hydroxy-3,6,7,8-Tetramet hoxyflavone isomer
156	25.64	445.1140	445.1144	0.88	$C_{22}H_{22}O_{10}$	MS^2[445]: 283.0614(100)		Oroxylin A-O-glucoside
157	25.69	821.3965	821.39783	1.61	$C_{42}H_{62}O_{16}$	MS^2[821]: 113.0232(100), 85.0282(92), 193.0351(32), 351.0582(26)		Glycyrrhizic acid isomer

158	25.77	471.2013	471.2015	0.29	$C_{26}H_{30}O_8$		MS^2[471]: 161.0596(100), 425.1955(33), 367.1908(11)	Limonin isomer
159	25.78	431.0984	431.0986	0.53	$C_{21}H_{20}O_{10}$	MS^2[431]: 283.0613(100), 239.0715(12), 269.0457(8)		Oroxylin A derivative
160	25.81	983.4493	983.4486	-0.73	$C_{48}H_{72}O_{21}$	MS^2[983]: 113.0233(100), 193.0350(29), 351.0584(28)		Licorice-saponin A3
161	25.85	389.1231	389.1229	-0.60	$C_{20}H_{20}O_8$		MS^2[389]: 359.0758(100), 169.0130(19), 197.0081(15), 113.0241(4)	3-Hydroxy-5,6,7,8,4'-Pentamethoxyflavanone isomer
162	26.04	837.3914	837.3922	0.94	$C_{42}H_{62}O_{17}$	MS^2[837]: 113.0232(100), 193.0346(30), 351.0569(20)		Licorice-saponin G2 isomer
163	26.06	359.1125	359.1122	-0.89	$C_{19}H_{18}O_7$		MS^2[359]: 329.0653(100), 286.0467(11),	5-Hydroxy-3,6,7,8-Tetramethoxyflavone isomer
164	26.17	389.1231	389.1230	-0.27	$C_{20}H_{20}O_8$		MS^2[389]: 359.0757(100)	3-Hydroxy-5,6,7,8,4'-Pentamethoxyflavanone isomer
165	26.48	879.4020	879.4034	1.58	$C_{44}H_{64}O_{18}$	MS^2[879]: 113.0233(100), 193.0347(23), 351.0572(18)		22-Acetoxyglycyrrhizin isomer
166	26.54	837.3914	837.3926	1.38	$C_{42}H_{62}O_{17}$	MS^2[837]: 113.0232(100), 193.0350(31), 351.0564(25)		Licorice-saponin G2 isomer

167	26.64	419.1337	419.1336	-0.12	$C_{21}H_{22}O_9$		MS^2[419]: 389.0864(100), 404.1103(11), 371.0753(9)	3-Hydroxy-5,6,7,8,3',4'-Hexamethoxyflavone isomer
168	27.13	819.3809	819.3818	1.11	$C_{42}H_{60}O_{16}$	MS^2[819]: 113.0232(100), 193.0347(28), 351.0572(21)		Licorice saponin E2 isomer
169	27.17	373.1282	373.1279	-0.67	$C_{20}H_{20}O_7$		MS^2[373]: 343.0809(100), 315.0857(16), 358.1042(10)	Isosinensetin isomer
170	27.33	367.1187	367.1188	0.16	$C_{21}H_{20}O_6$	MS^2[367]: 309.0409(100), 297.0402(16), 284.0311(12)		Glycycoumarin isomer
171	27.36	837.3914	837.3923	1.08	$C_{42}H_{62}O_{17}$	MS^2[837]: 113.0232(100), 193.0352(27), 351.0569(25)		Licorice-saponin G2 isomer
172	27.49	403.1387	403.1384	-0.85	$C_{21}H_{22}O_8$		MS^2[403]: 373.0914(100), 327.0856(47), 388.1141(8), 355.0818(6)	Nobiletin isomer
173	27.59	879.4020	879.4031	1.31	$C_{44}H_{64}O_{18}$	MS^2[879]: 113.0233(100), 193.0346(19), 351.0532(16)		22-Acetoxyglycyrrhizin isomer
174	27.71*	283.0612	283.0615	1.04	$C_{16}H_{12}O_5$	MS^2[283]: 268.0378(100), 163.0027(20), 239.0346(4), 211.0394(2)		Wogonin

175	27.71	285.0768	285.0769	0.19	$C_{16}H_{14}O_5$	MS^2[285]: 164.0107(100), 151.0027(46)		Sakuranetin isomer
176	27.77	837.3914	837.3918	0.43	$C_{42}H_{62}O_{17}$	MS^2[837]:113.0232(100), 193.0349(27), 351.0566(20)		Licorice-saponin G2 isomer
177	27.90	373.1282	373.1278	-1.07	$C_{20}H_{20}O_7$		MS^2[373]: 343.0809(100), 315.0859(34), 358.1044(14)	Isosinensetin isomer
178	28.00	863.4071	863.4080	1.10	$C_{44}H_{64}O_{17}$	MS^2[863]: 351.0573(100)		22β-acetoxylglycyrrhaldehyde isomer
179	28.15	283.0612	283.0614	0.61	$C_{16}H_{12}O_5$	MS^2[283]: 240.0455(100), 268.0367(2)		Oroxylin A isomer
180	28.29	837.3914	837.3921	0.80	$C_{42}H_{62}O_{17}$	MS^2[837]: 113.0232(100), 351.0568(28), 193.0345(10)		Licorice-saponin G2 isomer
181	28.33	403.1387	403.1382	-1.47	$C_{21}H_{22}O_8$		MS^2[403]: 373.0913(100), 3355.0808(21),327.0853(10), 88.1154(7)	Nobiletin isomer
182	28.39	389.1231	389.1226	-1.30	$C_{20}H_{20}O_8$		MS^2[389]: 359.0756(100)	3-Hydroxy-5,6,7,8,4'-Pentamethoxyflavanone isomer
183	28.73*	283.0612	283.0613	0.29	$C_{16}H_{12}O_5$	MS^2[283]: 239.0712(100), 238.0633(29), 240.0426(22)		Oroxylin A
184	28.74	373.1282	373.1279	-0.83	$C_{20}H_{20}O_7$		MS^2[373]: 343.0810(100), 358.1043(30), 315.0862(20)	Isosinensetin isomer

185	28.74	375.1074	375.1071	-0.94	$C_{19}H_{18}O_8$		MS^2[375]: 345.0598(100), 327.0494(43)	6,7,8,3',4'-Pentamethoxy flavanone isomer
186	28.96*	821.3965	821.3970	0.56	$C_{42}H_{62}O_{16}$	MS^2[821]: 113.0232(100), 85.0282(76), 193.0348(25), 351.0562(16)		Glycyrrhizic acid
187	29.08	837.3914	821.3973	0.94	$C_{42}H_{62}O_{17}$	MS^2[837]: 113.0233(100), 193.0348(27), 351.0583(20)		Licorice-saponin G2 isomer
188	29.12*	403.1387	403.1381	-1.70	$C_{21}H_{22}O_8$		MS^2[403]: 373.0915(100), 327.0853(10), 388.1141(8), 355.0804(5)	Nobiletin
189	29.15	343.1176	343.1170	-1.70	$C_{19}H_{18}O_6$		MS^2[343]: 313.0703(100), 285.0755(23),153.0181(18), 133.0647(8)	5,7,3',4'-Tetramethoxy flavone isomer
190	29.50	373.1282	373.1277	-1.23	$C_{20}H_{20}O_7$		MS^2[373]: 343.0809(100), 315.0859(8), 358.1044(8)	Isosinensetin isomer
191	29.51	863.4071	863.4078	0.89	$C_{44}H_{64}O_{17}$	MS^2[863]: 351.0576(100), 193.0349(18)		22β-acetoxylglycyrrhaldehyde isomer
192	29.57	821.3965	821.3973	0.94	$C_{42}H_{62}O_{16}$	MS^2[821]: 113.0232(100), 85.0282(80), 193.0348(27), 351.0572(17)		Glycyrrhizic acid isomer
193	29.61	433.1493	433.1488	-1.11	$C_{22}H_{24}O_9$		MS^2[433]: 403.1019(100),	3,5,6,7,8,3',4'-Heptamethoxyflavone isomer

							385.0909(11), 418.1254(8)	
194	29.73	343.1176	343.1173	-0.89	$C_{19}H_{18}O_6$		MS^2[343]: 313.0701(100), 285.0754(34),153.0182(22), 133.0648(13)	5,7,3',4'-Tetramethoxy flavone isomer
195	29.75	403.1387	403.1384	-0.85	$C_{21}H_{22}O_8$		MS^2[403]: 373.0914(100), 327.0858(12), 388.1151(10), 355.0811(6)	Nobiletin isomer
196	29.83	863.4071	863.4080	1.10	$C_{44}H_{64}O_{17}$	MS^2[863]: 113.0232(100), 193.0347(28), 351.0572(17)		22β-acetoxylglycyrrhaldehyde isomer
197	29.86	367.1187	367.1189	0.43	$C_{21}H_{20}O_6$	MS^2[367]: 309.0406(100), 297.0406(32), 284.0327(11)		Glycycoumarin isomer
198	29.88	821.3965	821.3974	1.09	$C_{42}H_{62}O_{16}$	MS^2[821]: 113.0232(100), 85.0282(89), 351.0575(25), 193.0348(23)		Glycyrrhizic acid isomer
199	30.20	823.4122	823.4130	1.05	$C_{42}H_{64}O_{16}$	MS^2[823]: 113.0232(100), 193.0343(28), 351.0575(28)		Licorice saponin J2 isomer
200	30.23	863.4071	863.4082	1.31	$C_{44}H_{64}O_{17}$	MS^2[863]: 351.0573(100)		22β-acetoxylglycyrrhaldehyde isomer
201	30.31	373.1282	373.1278	-1.07	$C_{20}H_{20}O_7$		MS^2[373]: 343.0809(100), 315.0864(19), 358.1061(9)	Isosinensetin isomer

202	30.81	821.3965	821.3975	1.23	$C_{42}H_{62}O_{16}$	MS^2[821]: 113.0232(100), 85.0282(79), 193.0349(32), 351.0567(27)		Glycyrrhizic acid isomer
203	30.86	373.1282	373.1277	-1.23	$C_{20}H_{20}O_7$		MS^2[373]: 343.0808(100), 358.1048(8), 315.0861(4)	Isosinensetin isomer
204	31.13	381.1344	381.1350	0.42	$C_{22}H_{22}O_6$	MS^2[381]: 323.0561(100), 351.0873(33), 311.0564(29)		Licoricone isomer
205	31.16	823.4122	823.4132	1.20	$C_{42}H_{64}O_{16}$	MS^2[823]: 113.0232(100), 193.0349(28), 351.0580(26)		Licorice saponin J2 isomer
206	31.19	805.4016	805.4026	1.24	$C_{42}H_{62}O_{15}$	MS^2[805]: 351.0572(100)		Licorice saponin C2 isomer
207	31.47	403.1387	403.1385	-0.71	$C_{21}H_{22}O_8$		MS^2[403]: 373.0914(100), 355.0808(11), 388.1151(10), 327.0856(3)	Nobiletin isomer
208	31.61	389.1231	389.1229	-0.60	$C_{20}H_{20}O_8$		MS^2[389]: 359.0757(100), 169.0130(22), 197.0080(21), 113.0238(4)	3-Hydroxy-5,6,7,8,4'-Pentamethoxyflavanone isomer
209	31.75	823.4122	823.4131	1.13	$C_{42}H_{64}O_{16}$	MS^2[823]:113.0232(100), 351.0578(36), 193.0336(34)		Licorice saponin J2 isomer
210	31.83	821.3965	821.3977	1.46	$C_{42}H_{62}O_{16}$	MS^2[821]: 113.0233(100), 85.0281(97),		Glycyrrhizic acid isomer

						351.0580(30), 193.0347(30)		
211	31.86*	593.1301	593.1305	0.72	$C_{30}H_{26}O_{13}$	MS^2[593]: 209.0450(100), 121.0283(10)		Proanthocyanidins
212	32.15*	269.0455	269.0456	0.27	$C_{15}H_{10}O_5$	MS^2[269]: 225.0553(100), 241.0504(33)		Emodin
213	32.25	389.1231	389.1228	-0.68	$C_{20}H_{20}O_8$		MS^2[389]: 359.0757(100), 169.0121(2)	3-Hydroxy-5,6,7,8,4'-Pentamethoxyflavanone isomer
214	32.56	823.4122	823.41266	0.61	$C_{42}H_{64}O_{16}$	MS^2[823]: 113.0231(100), 193.0349(34), 351.0569(32)		Licorice saponin J2 isomer
215	32.59	365.1031	365.1032	0.24	$C_{21}H_{18}O_6$	MS^2[365]: 307.0249(100), 295.0248(42)		Glycyrol isomer
216	32.88	805.4016	805.4026	1.24	$C_{42}H_{62}O_{15}$	MS^2[805]: 113.0232(100), 351.0382(33), 193.0388(20)		Licorice saponin C2 isomer
217	33.05	351.0874	351.0876	0.594	$C_{20}H_{16}O_6$	MS^2[351]: 283.0977(100), 199.07756(41), 241.0866(31), 265.0871(21)		Licoisoflavone B isomer
218	33.15	359.1125	359.1123	-0.72	$C_{19}H_{18}O_7$		MS^2[359]: 329.0652(100), 286.0460(12)	5-Hydroxy-3,6,7,8-Tetramet hoxyflavone isomer
219	33.23	805.4016	805.4027	1.31	$C_{42}H_{62}O_{15}$	MS^2[805]: 351.0567(100)		Licorice saponin C2 isomer
220	34.69	283.0612	283.0613	0.40	$C_{16}H_{12}O_5$	MS^2[283]: 255.0662(100), 239.0710(51),		Oroxylin A isomer

						241.0506(24), 211.0760(13)	
221	34.95	283.0612	283.0613	0.29	$C_{16}H_{12}O_5$	MS^2[283]: 239.0710(100), 255.0662(79), 224.0471(11)	Oroxylin A isomer
222	40.04*	455.3531	455.3526	-1.03	$C_{30}H_{48}O_3$	MS^2[455]: 391.2349(100), 409.2440(16), 349.8991(10), 308.9463(10)	Oleanic acid

* With standard references.

Supplementary Table S2: Detailed information of the 40 standards in Tojapride

Name	Formula	Purity	Article number	Company
Liquiritin	$C_{21}H_{22}O_9$	HPLC＞98%	114905186	Sigma-aldrich
Isoliquiritin	$C_{15}H_{10}O_6$	HPLC＞98%	104288219	Sigma-aldrich
Orientin	$C_{21}H_{20}O_{11}$	HPLC＞98%	H-044-181025	Chengdu Herpurify Co.,Ltd
Luteolin 7-O-β-D-glucoside	$C_{21}H_{20}O_{11}$	HPLC＞98%	M-025-181025	Chengdu Herpurify Co.,Ltd
Luteolin	C15H10O6	HPLC＞98%	M-007-190422	Chengdu Herpurify Co.,Ltd
Calycosin	$C_{16}H_{12}O_5$	HPLC＞98%	M-021	Chengdu Herpurify Co.,Ltd

Wogonin	$C_{16}H_{12}O_5$	HPLC＞98%	98817832	Sigma-aldrich
Oroxylin A	$C_{16}H_{12}O_5$	HPLC＞98%	Q-020	Chengdu Herpurify Co.,Ltd
Kaempferol 3-O-neohesperidoside	$C_{27}H_{30}O_{15}$	HPLC＞98%	CFS201901	Wuhan chemfaces biochemical company
Nicotiflorin	$C_{27}H_{30}O_{15}$	HPLC＞98%	CFN99830	Wuhan chemfaces biochemical company
Hyperoside	$C_{21}H_{20}O_{12}$	HPLC＞98%	111521-201507	National Institutes for Food and Drug Control
Isoquercitrin	$C_{21}H_{20}O_{12}$	HPLC＞98%	Y-076-181025	Chengdu Herpurify Co.,Ltd
Rutin	$C_{27}H_{30}O_{16}$	HPLC＞98%	AF8032520	Chengdu Alfa Biotechnology Co.,Ltd.
Baicalin	$C_{21}H_{18}O_{11}$	HPLC＞98%	DST190312-024	Chengdu desite biological technology co lt d
Hesperidin	$C_{28}H_{34}O_{15}$	HPLC＞98%	MUST-14081916	Chengdu mustbio-technology Co.,Ltd
Vitexin	$C_{15}H_{10}O_5$	HPLC＞98%	M-023-181025	Chengdu Herpurify Co.,Ltd
Quercetin	$C_{15}H_{10}O_7$	HPLC＞98%	C28J11Y116820	Shanghai yuanye Bio-Technology Co., Ltd
Eriodictyol	$C_{15}H_{12}O_6$	HPLC＞98%	PS1160-0025	Chengdu Push Bio-technology Co.,Ltd
Naringenin	$C_{15}H_{12}O_5$	HPLC＞98%	YJ0603HA13	Shanghai yuanye Bio-Technology Co., Ltd

Nobiletin	$C_{21}H_{22}O_8$	HPLC＞98%	C-015	Chengdu Herpurify Co.,Ltd
Aloe emodin	$C_{15}H_{10}O_5$	HPLC＞98%	PCSO253	Chengdu HerbSubstance Co.,Ltd
Emodin	$C_{15}H_{10}O_5$	HPLC＞98%	PCSO247	Chengdu HerbSubstance Co.,Ltd
Proanthocyanidins	$C_{30}H_{26}O_{13}$	HPLC＞98%	wkq19050610	Sichuan Weikeqi Biotechnology Co., Ltd.
Magnoflorine	$C_{20}H_{24}NO_4^+$	HPLC＞98%	DST200519-004	Chengdu Lemeitian Pharmaceutical Technology Co., Ltd.
Chlorogenic acid	$C_{16}H_{18}O_9$	HPLC＞98%	L-007-171216	Chengdu Herpurify Co.,Ltd
Cryptochlorogenic acid	$C_{16}H_{18}O_9$	HPLC＞98%	Y-067-180425	Chengdu Herpurify Co.,Ltd
Caffeic acid	$C_9H_8O_4$	HPLC＞98%	K-003	Chengdu Herpurify Co.,Ltd
Isoferulic acid	$C_{10}H_{10}O_4$	HPLC＞98%	11698-201103	National Institutes for Food and Drug Control
Azelaic acid	$C_9H_{16}O_4$	HPLC＞98%	R-064	Chengdu Herpurify Co.,Ltd
Octanedioic acid	$C_8H_{14}O_4$	HPLC＞98%	X-099	Chengdu Herpurify Co.,Ltd
Malic Acid	$C_4H_6O_5$	HPLC＞98%	P-013	Chengdu Herpurify Co.,Ltd

Salicylic acid	$C_7H_6O_3$	HPLC＞98%	H31A10B96399	Shanghai yuanye Bio-Technology Co., Ltd
Citraconic acid	$C_5H_6O_4$	HPLC＞98%	N-060	Chengdu Herpurify Co.,Ltd
Gallic acid	$C_7H_6O_5$	HPLC＞98%	PCS0637	Chengdu HerbSubstance Co.,Ltd
Protocatechuic acid	$C_{15}H_{20}O_4$	HPLC＞98%	Y-031	Chengdu Herpurify Co.,Ltd
Abscisic acid	$C_{15}H_{20}O_4$	HPLC＞98%	T-048	Chengdu Herpurify Co.,Ltd
Quinic acid	$C_7H_{12}O_6$	HPLC＞98%	A06N11L130218	Shanghai yuanye Bio-Technology Co., Ltd
Methyl gallate	$C_8H_8O_5$	HPLC＞98%	PCS0634	Chengdu HerbSubstance Co.,Ltd
Glycyrrhizic acid	$C_{42}H_{62}O_{16}$	HPLC＞98%	100486660	Sigma-aldrich
Oleanic acid	$C_{30}H_{48}O_3$	HPLC＞98%	110709-201206	National Institutes for Food and Drug Control
